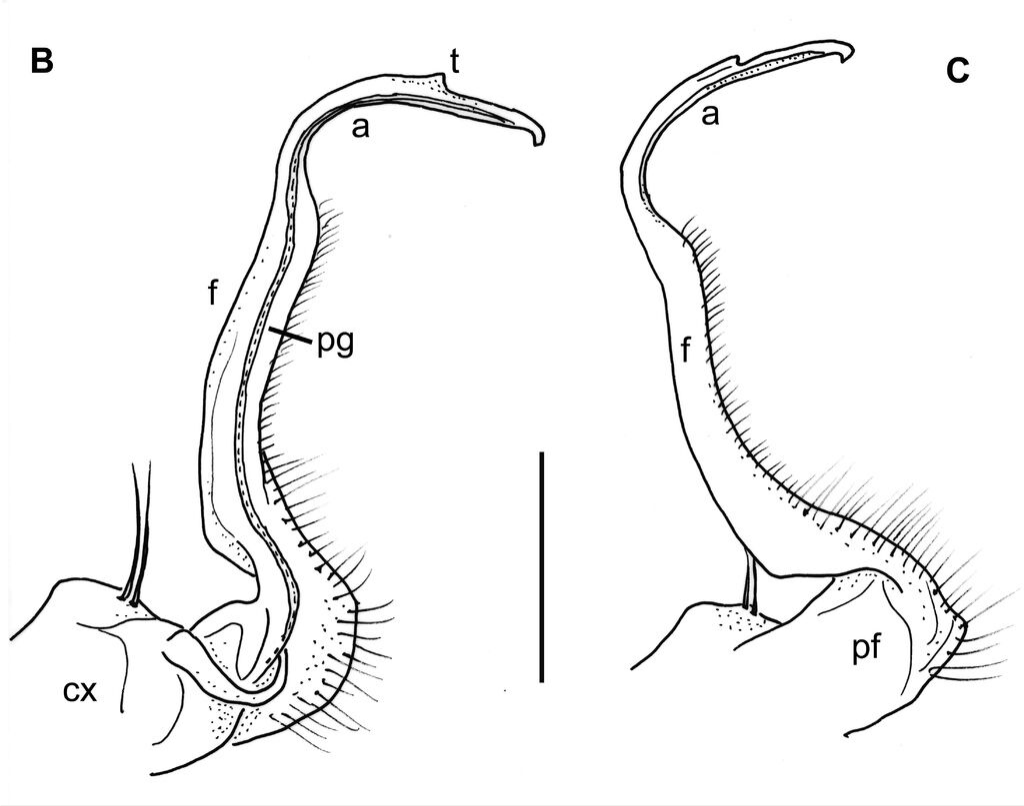# First description of a male *Melapheapamea* Hoffman & Lohmander, 1968 from Turkey (Diplopoda, Polydesmida, Xystodesmidae)

**DOI:** 10.3897/BDJ.13.e156970

**Published:** 2025-05-16

**Authors:** Zoltán Korsós, Sergei Golovatch

**Affiliations:** 1 University of Veterinary Medicine Budapest, Budapest, Hungary University of Veterinary Medicine Budapest Budapest Hungary; 2 Hungarian Natural History Museum, Budapest, Hungary Hungarian Natural History Museum Budapest Hungary; 3 Institute of Ecology and Evolution, Russian Academy of Sciences, Moscow, Russia Institute of Ecology and Evolution, Russian Academy of Sciences Moscow Russia

**Keywords:** millipede, male specimen, Turkey

## Abstract

**Background:**

The male of *Melapheapamea* Hoffman & Lohmander, 1968 is described and illustrated for the first time, based on a single specimen collected by a Hungarian expedition to Turkey in 1980.

**New information:**

The specimen is kept in the Myriapoda Collection of the Hungarian Natural History Museum (Hungarian National Museum Public Collection Centre), Budapest.

## Introduction

The millipede genus *Melaphe* Cook, 1904 in the subfamily Melaphinae (Brölemann, 1916) was initially reviewed in the modern sense by Hoffman (1962), who recognised only two species: *Melaphecorrupta* Attems, 1944 and *M.vestita* (C. L. Koch, 1847) with its nominal form and two subspecies, *M.vestitavestita* and *M.vestitathracia* (Verhoeff, 1926). In a later revision, Hoffman and Lohmander (1968) not only added *Melaphemauritanica* (Lucas, 1844) and *M.cypria* (Humbert & de Saussure, 1869) to the genus, but they also described two new congeners: *M.apamea* Hoffman & Lohmander, 1968 and *Melaphecastianeira* Hoffman & Lohmander, 1968. A seventh species, *M.albanica* Verhoeff, 1932 had already been transferred to the new genus *Ochridaphe* Hoffman, 1962 in the earlier paper by Hoffman (1962). Marek et al. (2014) also listedthese six *Melaphe* species in their Xystodesmidae catalogue. There are two more possible *Melaphe* species, but their status is uncertain: *M.blainvillii* (Eydoux & Gervais, 1836) and *M.thrax* (Brandt, 1839).

The species *Melapheapamea* was described, based on female specimens only. The types were deposited in the Entomological Collection of the Zoological Institute and Zoological Museum of the University of Hamburg (now MNHG) (Rack 1974).

During a visit in 1981 to the HNHM, Budapest, the second author of the present paper (SG) examined material collected in Turkey and found a single (broken) male specimen of *Melapheapamea*. The description was not published and the record was only mentioned later in the publication by Enghoff (2006). In the present paper, we make up for this omission and provide a detailed description of the single male specimen of *M.apamea* known to date.

## Materials and methods

The specimen is kept in the Myriapoda Collection of the HNHM, in 70% ethanol (Fig. 1a). It was examined and labelled first by SG (Fig. 1b) in 1981; then described and illustrated by ZK using a Leica M125 stereomicroscope. Gonopods were removed and placed into a separate microvial. Photographs were also taken by ZK with a Samsung A30 mobile phone.

### Abbreviations:

HNHM = Hungarian Natural History Museum (Hungarian National Museum Public Collection Centre), Budapest, Hungary

MNHG = Museum of Nature Hamburg, Germany

## Taxon treatments

### Melaphe

Cook, 1904

5E18ED07-70A7-5193-A1D2-761CA28A1682

https://www.millibase.org/aphia.php?p=taxdetails&id=892003

Oxyurus vestitus C. L. Koch, 1847Cook (1904): 55. Preoccupied and replaced

#### Description

##### Diagnosis

The genus is characterised by the retention of a distinct sternite between the gonocoxae and by various details of gonopodal structure. Coxite simple, without apophyses; telopodites long and slender, *in situ* crossing each other distally, each arcuately curved anteroventrad, with neither prefemoral nor femoral processes (Hoffman 1962).

##### Distribution

Mediterranean Region, North Africa and Near East (Morocco, Algeria, Cyprus and Turkey), but one species in Ethiopia.

### Melaphe apamea

Hoffman & Lohmander, 1968

04A8E8F8-16E3-5CB9-AF2E-2D9F2FDAD927

https://www.millibase.org/aphia.php?p=taxdetails&id=937275

*Melapheapamea* Hoffman & Lohmander, 1968: 109-110, figs. 49, 50.
*M.apamea* Enghoff 2006: 189.

#### Materials

- **Type status:** Holotype. **Occurrence:** occurrenceID: 67E83CBC-6484-573C-B58A-CB772FFC18A0; **Location:** continent: Asia; country: Turkey; countryCode: TR; municipality: Dinar; verbatimCoordinates: 38 04N 30 10E; **Identification:** identificationID: female; identifiedBy: Richard L. Hoffman & H. Lohmander; **Event:** eventID: collection; eventDate: May 1955; year: 1955; month: 5; eventRemarks: leg. H. Couffait; **Record Level:** institutionID: Museum of Nature Hamburg, Germany; collectionID: ZMH-ARA-A0029195; institutionCode: MNHG; basisOfRecord: PreservedSpecimen
- **Type status:** Paratype. **Occurrence:** occurrenceID: 42A0972B-C72E-54E7-AA9D-1FA5ADEA3611; **Location:** continent: Asia; country: Turkey; countryCode: TR; municipality: Dinar; verbatimCoordinates: 38 04N 30 10E; **Identification:** identifiedBy: Richard L. Hoffman & H. Lohmander; **Event:** eventID: collection; eventDate: May 1955; year: 1955; month: 5; eventRemarks: leg. H. Couffait; **Record Level:** institutionID: Museum of Nature Hamburg, Germany; collectionID: ZMH-ARA-A0029196; institutionCode: MNHG; basisOfRecord: PreservedSpecimen
- **Type status:** Paratype. **Occurrence:** occurrenceID: 64839FF7-42F2-5F8A-B884-C92E5724ABB0; **Location:** continent: Asia; country: Turkey; countryCode: TR; municipality: near Denizli; locality: Dodurgaköyü Cave; **Identification:** identificationID: female; identifiedBy: Richard L. Hoffman & H. Lohmander; **Event:** eventID: collection; eventDate: Nov. 1946; year: 1946; month: 11; eventRemarks: leg. C. Kosswig; **Record Level:** institutionID: Museum of Nature Hamburg, Germany; collectionID: ZMH-ARA-A0029196; institutionCode: MNHG; basisOfRecord: PreservedSpecimen
- **Type status:** Other material. **Occurrence:** occurrenceID: 6A9FEEFE-1348-571D-B893-3877590D8483; **Location:** continent: Asia; country: Turkey; countryCode: TR; stateProvince: Antalya; municipality: Beskonak; locality: Toros Mts, 15 km north of Beskonak; verbatimElevation: 1500 m a.s.l.; locationRemarks: soil sample collected in *Cedrus*-*Cupressus* forest; **Identification:** identificationID: male; identifiedBy: Sergei I. Golovatch; **Event:** eventID: collection; samplingProtocol: soil sample; eventDate: 10. Oct. 1980; year: 1980; month: 10; day: 10; habitat: *Cedrus*-*Cupressus* forest; eventRemarks: leg. L. Peregovits

#### Description

The posterior part of the single male specimen is broken-off; it has only 14 body rings (Fig. 1a).

The length of broken part is only 30 mm, mid-body width at ring 10 with paranota, 5.5 mm, metatergal length, 2.1 mm, collum width, 5.3 mm, median collum length, 2.1 mm.

Colour in ethanol completely faded, yellowish-white, no pattern traceable (Fig. 1).

Head smooth, epicranial suture distinct, 2+2 frontal setae, 1+1 pre-antennal setae, 1+1 inter-antennal setae, a row of short supra-labral setae.

Antennae straight, slender, antennomere 1 globose; antennomeres 2–6 elongated, subequal in length, antennomere 7 smaller than previous ones, rectangular, with 4 apical sensory cones. Collum subtrapezoid in shape, convex, 2 times as long as metatergum 2, anterior margin straight, without ridge, posterior margin undulated.

**Figure 2.**
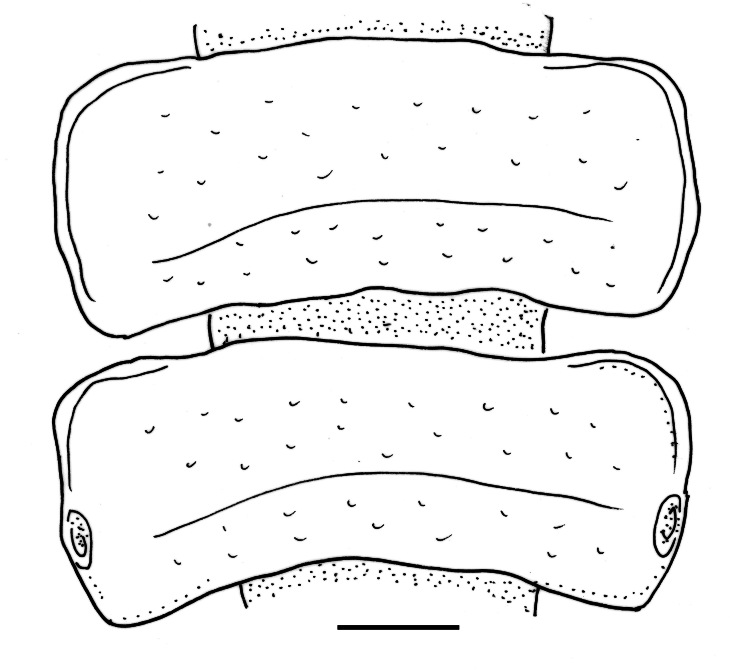
9^th^ and 10^th^ segments of *Melapheapamea* ♂, dorsal view. Scale bar 1 mm.

Rings 2–5 narrower than following ones, rings 6–14 parallel-sided, last rings missing. Proterga completely smooth, metaterga very weakly striolate, each with a pale transverse depression in the middle (Fig. 2). All rings with weak anterior and lateral ridges, posterolateral corner only very weakly pointed, almost rounded. Pore formula normal, pores on rings 5, 7, 9, 10, 12 and 13, all dorsal and located near lateral margins of paranota. Terminal rings (15–20) missing, but, according to Hoffman and Lohmander (1968): 110), epiproct, paraprocts and hypoproct without pecularities.

Bases of mid-body leg-pairs well separated, sterna smooth and wide. Coxa stout, as long as wide; prefemur twice as long as coxa, without prefemoral spine, but ventrally with a single distal seta; femur slender, about twice as long as prefemur, no femoral pads; postfemur shot, stout, similar to prefemur; tibia slender, about 1.2 times as long as postfemur; tarsus more slender, about 1.2 times as long as tibia, densely setose; claws normal (Fig. 3b).

**Figure 4a.**
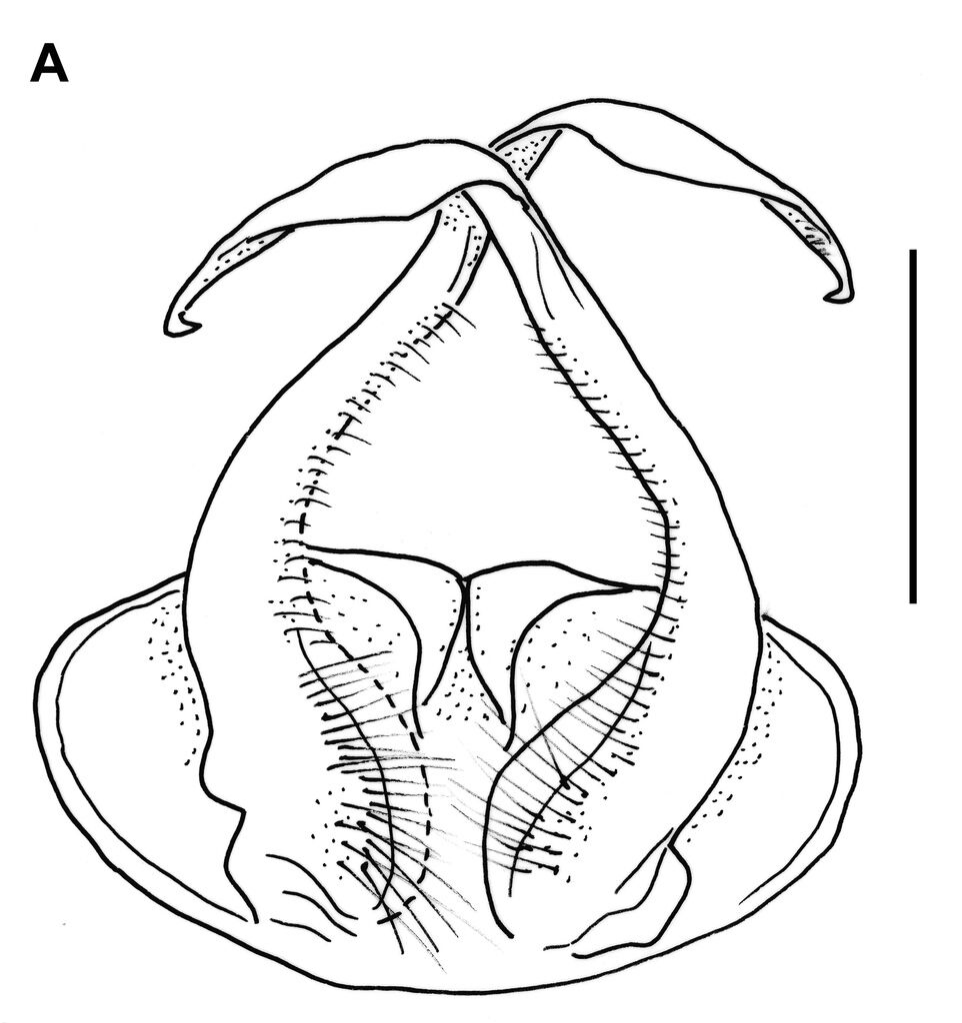


Male characters: Coxae of second leg-pair each with a small, triangular coxal process or gonapophysis (cxp, Fig. 3a). Gonopods barely divided into a coxite and a telopodite (Fig. 4). Coxite (cx) strong, stout, without apophyses, with two long setae on its anterior side; prefemorite (pf) about twice as long as wide, usually densely setose, slightly curved dorsad; femorite (f) slender, straight, with a number of short setae along its ventral margin; acropodite (a) slender, bent ventromesad at a perfect right angle, its length about half of femorite, at its mid-way with a small tooth (t) on a slightly twisted and flattened flap; tip of acropodite small, turned backwards; prostatic groove (pg) running mesad in the middle of a whole gonopod, opening just in front of a curved tip of acropodite.

Female characters: The species was originally described from females (Hoffman and Lohmander 1968), whose somatic characters are in agreement with the present male. However, no specific female characters, such as cyphopods, were mentioned in the description. Without examining the types or new material, we cannot add new information.

##### Diagnosis

A moderate-sized species of *Melaphe*, easily recognisable by the larger paranota which are set high and nearly horizontal; dorsum of metazonites with distinct scattered tubercles especially on the bases of the paranota (Hoffman and Lohmander 1968). Male coxae of second leg-pair with small coxal processes (= gonapophyses); gonopods similar to *M.vestita*, but femorite straight and slender, without lobes, acropodite with a small tooth at mid-length.

#### Taxon discussion

The first male of the species has been identified and described.

## Discussion

The specimen descibed here is the first male examined of the species. In their Xystodesmidae catalogue, Marek et al. (2014: 84)) mistakenly referred to the holotype of *Melapheapamea* as a male; although Enghoff (2006: 189)) already pointed out that the specimen in HNHM “is the first male ever recorded”.

Hoffman and Lohmander (1968) provided a key to the six species of *Melaphe* recognised at that time, based on males, except for *M.apamea*, which was incorporated therein using only the shape and structure of metaterga. Enghoff (1995), comparing the geography of several Turkish millipedes, presented a cladogram for six species of *Melaphe*, where *M.apamea* (with question mark) joined with two others (*M.vestita* and *M.castianeira*) representing western Turkey as the top candidate for the ancestral area of the genus (Enghoff 1995: 783, fig. 141).

As regards the present knowledge of the locations of specimens, it seems that *M.apamea* is confined to the south-central part of Turkey, mainly in and around Antalya Province.

## Supplementary Material

XML Treatment for Melaphe

XML Treatment for Melaphe apamea

## Figures and Tables

**Figure 1a.**
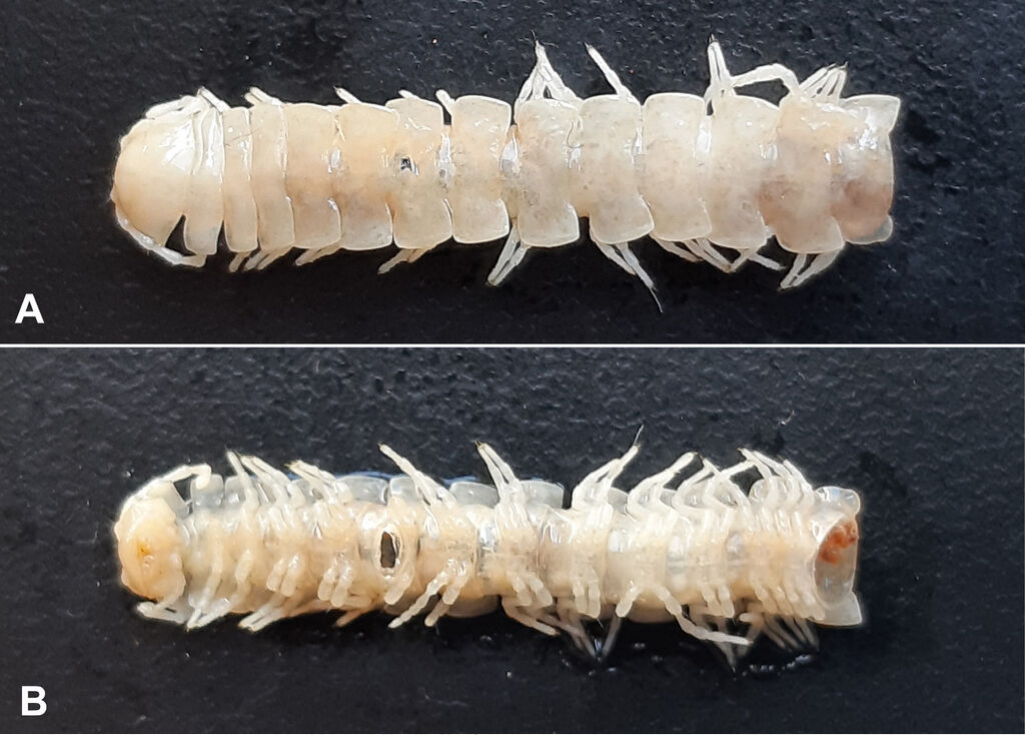


**Figure 1b.**
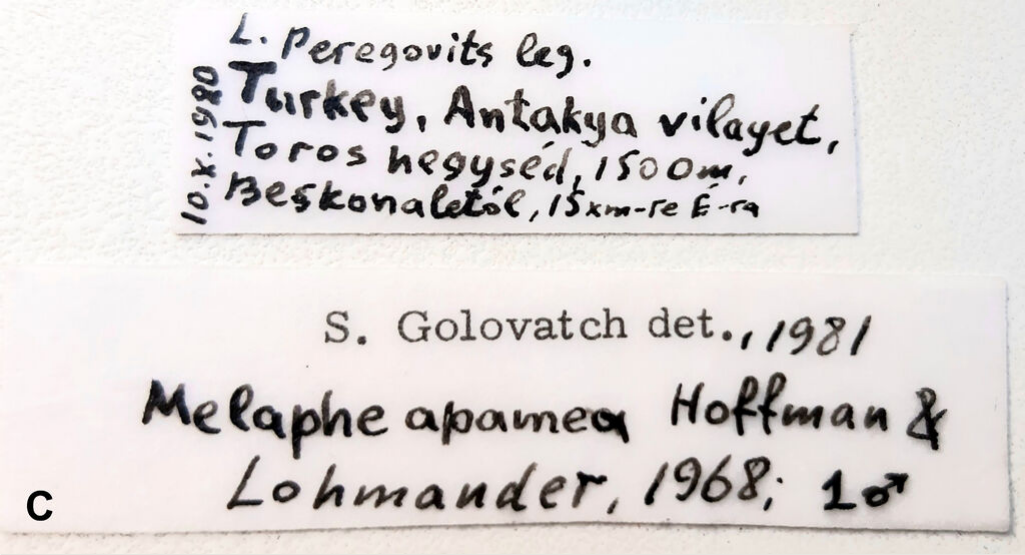


**Figure 3a.**
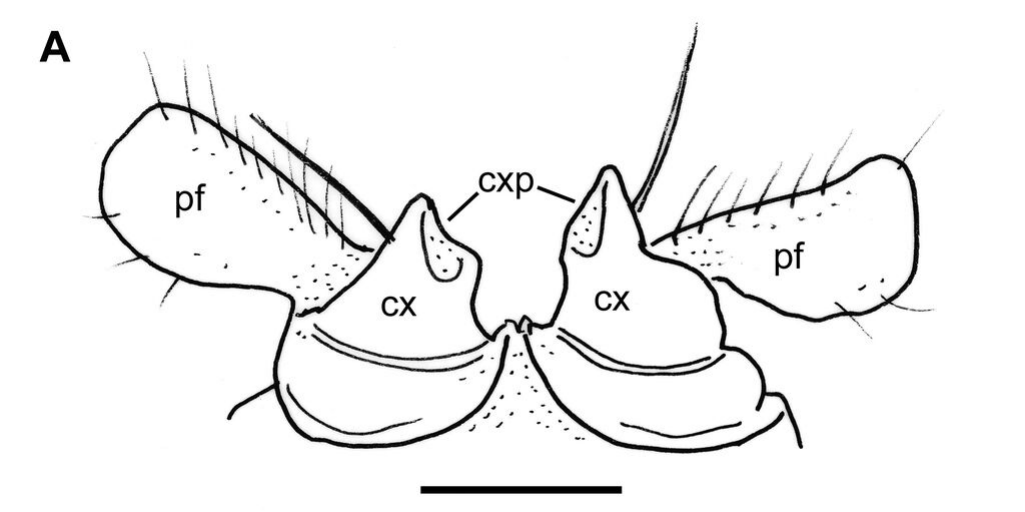


**Figure 3b.**
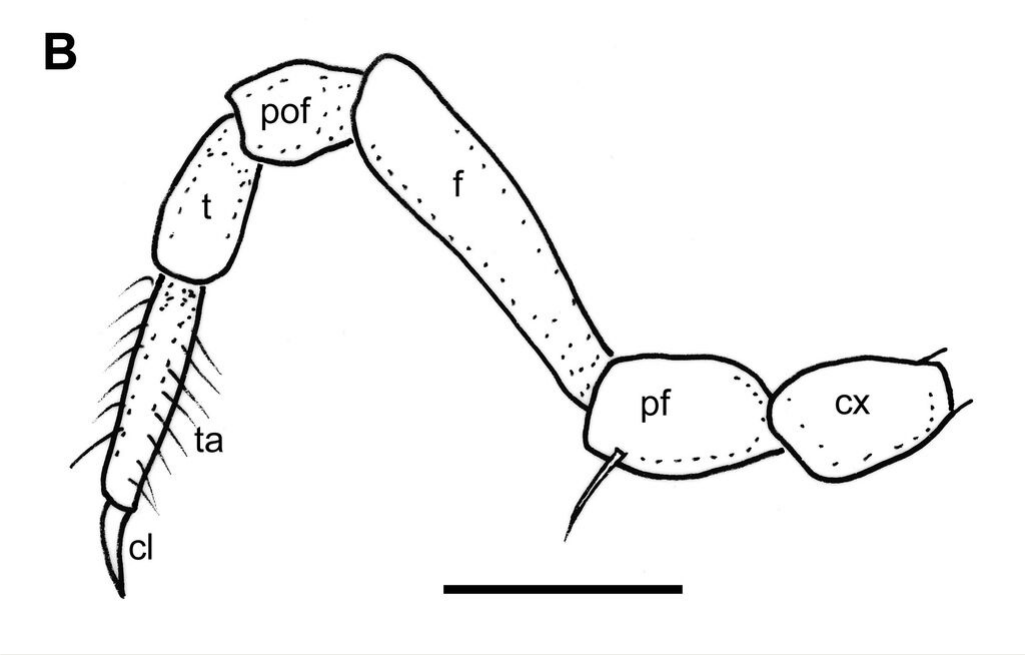


**Figure 4b.**